# Brazilian Group of Gastrointestinal Tumours’ consensus guidelines for the management of gastric cancer

**DOI:** 10.3332/ecancer.2020.1126

**Published:** 2020-10-20

**Authors:** Renata D’Alpino Peixoto, Duilio R Rocha-Filho, Rui F Weschenfelder, Juliana F M Rego, Rachel Riechelmann, Anelisa K Coutinho, Gustavo S Fernandes, Alexandre A Jacome, Aline C Andrade, Andre M Murad, Celso A L Mello, Diego S C G Miguel, Diogo B D Gomes, Douglas J Racy, Eduardo D Moraes, Eduardo H Akaishi, Elisangela S Carvalho, Evandro S Mello, Fauze Maluf Filho, Felipe J F Coimbra, Fernanda C Capareli, Fernando F Arruda, Fernando M A C Vieira, Flavio R Takeda, Guilherme C C Cotti, Guilherme L S Pereira, Gustavo A Paulo, Héber S C Ribeiro, Laercio G Lourenco, Marcela Crosara, Marcelo G Toneto, Marcos B Oliveira, Maria de Lourdes Oliveira, Maria Dirlei Begnami, Nora M Forones, Osmar Yagi, Patricia Ashton-Prolla, Patricia B Aguillar, Paulo C G Amaral, Paulo M Hoff, Raphael L C Araujo, Raphael P Di Paula Filho, Rene C Gansl, Roberto A Gil, Tulio E F Pfiffer, Tulio Souza, Ulysses Ribeiro Jr., Victor Hugo F Jesus, Wilson L Costa Jr, Gabriel Prolla

**Affiliations:** 1 Grupo Oncoclínicas, Brazil, 04543-906; 2 Rede D’Or, Brazil, 04535-110; 3 Hospital Moinhos de Vento, Porto Alegre, Brazil, 90035-000; 4 Hospital Universitário Onofre Lopes, Natal, Brazil, 59012-300; 5 AC Camargo Cancer Center, São Paulo, Brazil, 01525-001; 6 Clínica AMO, Salvador, Brazil, 41.950-640; 7 Hospital Sírio Libanês, Brazil, 01308-050; 8 Laboratório Personal, Belo Horizonte, Brazil; 9 Hospital Israelita Albert Einstein, São Paulo. Brazil, 05652- 900; 10 Hospital Beneficência Portuguesa de São Paulo, São Paulo, Brazil, 01323-001; 11 Faculdade de Medicina da Universidade de São Paulo, São Paulo, Brazil, 01246903; 12 Hospital São Rafael, Salvador, Brazil, 41253-190; 13 Américas Oncologia, Brazil, 22775-001; 14 Centro de Oncologia do Paraná, Curitiba, Brazil, 81200-100; 15 Universidade Federal de São Paulo, São Paulo, Brazil, 04040-003; 16 Hospital São Lucas da PUCRS, Porto Alegre, Brazil, 90610-000; 17 Faculdade de Ciências Médicas da Santa Casa de São Paulo, Sâo Paulo, Brazil, 01238-010; 18 Hospital de Clínicas de Porto Alegre, Porto Alegre, Brazil, 90035-903; 19 Hospital Alemão Oswaldo Cruz, São Paulo, Brazil, 01323-020; 20 Hospital Aliança de Salvador, Salvador, Brazil, 41920-900

**Keywords:** gastric cancer, gastroesophageal cancer, guidelines

## Abstract

Gastric cancer is among the ten most common types of cancer worldwide. Most cases and deaths related to the disease occur in developing countries. Local socio-economic, epidemiologic and healthcare particularities led us to create a Brazilian guideline for the management of gastric carcinomas. The Brazilian Group of Gastrointestinal Tumors (GTG) invited 50 physicians with different backgrounds, including radiology, pathology, endoscopy, nuclear medicine, genetics, oncological surgery, radiotherapy and clinical oncology, to collaborate. This document was prepared based on an extensive review of topics related to heredity, diagnosis, staging, pathology, endoscopy, surgery, radiation, systemic therapy and follow-up, which was followed by presentation, discussion, and voting by the panel members. It provides updated evidence-based recommendations to guide clinical management of gastric carcinomas in several scenarios and clinical settings.

## Introduction

Tumours of the upper gastrointestinal tract have high lethality rates and are among the worst 5-year survival estimates among any neoplasm. The disease is well known for its capability of spreading, with metastatic lesions, even at the early stages of the disease [41]. The Brazilian National Cancer Institute (INCA) estimated 13,540 new cases of gastric cancer in men and 7,750 in women in Brazil for 2018 and 2019. The incidences correspond to an estimated risk of 13.11 new cases in 100 thousand men and 7.32 in 100 thousand women [54]. Complete tumour resection remains the only available curative therapy, with evidence of increased survival if managed with adjuvant or perioperative treatment [27, 72, 97]. Treatment improvements over the past decade are moving towards an increasingly personalised approach aiming to ensure symptom control, improve patients’ quality of life and increase the survival of patients with gastric cancer.

## Objective

This report was put together by the Brazilian Group of Gastrointestinal Tumours (GTG), intending to express the opinion of Brazilian specialists regarding specific subjects related to the diagnosis, staging and treatment of gastric cancer. This document was prepared based on an extensive review of each item, followed by the panel members’ presentation, discussion and voting. The board specialists carefully revised this final report; however, the GTG emphasises that the judgment of the physician must prevail as the patient individuality is a critical aspect of in-person medical examination.

## Panel composition

A panel of 50 physicians with different backgrounds was invited to collaborate. Specialty fields included radiology, pathology, endoscopy, nuclear medicine, genetics, oncological surgery, radiotherapy and clinical oncology. All experts are involved in the practice of the diagnosis and treatment of patients with gastric cancer. Considering Brazil’s regional diversity, the GTG invited specialists from various regions, including representatives from several states (Bahia, Ceará, Rio Grande do Norte, Federal District, Minas Gerais, Rio Grande do Sul, Paraná, Rio de Janeiro and São Paulo). All members of the panel followed the recommendations of the Federal Council of Medicine concerning disclosure of potential conflicts of interest.

## Methodology

Topics were chosen by the GTG’s board of directors and distributed among members a few months in advance of in-person presentation and voting. All specialists did a comprehensive literature review, following the GTG’s standard guidelines to focus on each topic. All panel members were instructed to explain the level of evidence and strength of recommendation, as shown in Table 1. During the face-to-face meeting, the participants presented a summary of each of the reviewed topics, followed by discussion and voting when the opinions did not obtain consensus. In this report, experts’ views in different subjects may help to guide the therapeutic management of patients with gastric cancer in several stages of the disease and clinical settings. In specific topics, the recommendation may point out the absence of unanimity about the level of evidence or strength of recommendation. In this case, suggestions are described for each situation.

### Disclaimer

All recommendations were constructed upon scientific evidence. Readers must contextualise each recommendation according to the availability and access of the technologies in their clinical routine.

### Heredity

#### When to suspect hereditary syndromes in gastric cancer and how to evaluate patients

Approximately 1%–3% of all patients with gastric cancer have a hereditary predisposition to the disease, caused by a pathogenic germline variant in a cancer predisposition gene [86]. Only a few genotype–phenotype correlations have been established, and therefore, there is significant overlap in the phenotype associated with different genetic causes of gastric cancer. For this reason, genetic investigation of suspected cases should include comprehensive genetic testing using multigene panels. Table 2 summarises the main genes and clinical conditions associated with hereditary gastric cancer. Excellent reviews are available on this topic [109, 114].

In general, suspicion of hereditary cancer should be raised in the following situations [48]:

- Early onset of gastric cancer, if one of the diffuse types, diagnosed at or before the age of 40 in a patient or a relative, or diagnosis before the age of 50 with one close relative* with gastric cancer or a gastric cancer at any age with two or more close relatives affected with gastric cancer;
- Gastric cancer and a family history of cancer suggestive of a hereditary cancer syndrome (see Table 2);
- Gastric cancer and polyposis of the GI tract;
- Gastric cancer and another synchronous or metachronous primary solid tumour in the same patient;
- Presence of a germline pathogenic variant in a gastric cancer predisposition gene in a close relative.

(*) close relative = first- and second-degree relative.

- If a patient is suspected of having hereditary gastric cancer, he/she should be referred to a genetic risk evaluation by a specialist in cancer genetics. EVIDENCE LEVEL V, RECOMMENDATION A.

Results should also be discussed by genetics specialist and guidance to be offered.

The investigation of suspected cases of hereditary gastric cancer syndromes should consider the following premises:

1. Pre-test genetic counselling should be provided to all individuals and should include a discussion about potential risks, benefits and limitations of genetic testing, including a discussion of the possibility of detection of variants of uncertain significance. Whenever possible, results of genetic testing and guidance should be discussed by genetics specialist;
2. Except for selected situations in which the clinical diagnosis of the hereditary condition is obvious (i.e., Peutz–Jeghers syndrome) and single gene testing can be considered, most cases require molecular investigation using multigene panel testing;
3. In case a germline pathogenic variant in a cancer predisposition gene is identified, relatives should be informed of this result and cascade genetic testing of at-risk relatives should be recommended. The most efficient strategy to identify a causative gene mutation in a family is to test a close relative with cancer. If the relative is either unwilling or unavailable for testing, then consider testing of an unaffected relative.

### Staging

#### What tests are necessary for the diagnosis and staging of gastric cancer?

The following tests are considered necessary for the diagnosis and staging of gastric cancer.

- Upper digestive endoscopy (UDE) with biopsy when indicated. EVIDENCE LEVEL IV, RECOMMENDATION A.
- Computed tomography (CT) of the chest and abdomen with oral and intravenous contrast (not necessary if PET/CT is already carried out). EVIDENCE LEVEL II, RECOMMENDATION A.
- If CT is not clinically indicated, magnetic resonance imaging (MRI) should be carried out. EVIDENCE LEVEL II, RECOMMENDATION B.
- Echoendoscopy (in the absence of M1 disease) with a fine needle biopsy in the absence of clear signs of T > 2 or the presence of positive lymph nodes on CT. EVIDENCE LEVEL II, RECOMMENDATION A.
- CT of the pelvis, if clinically indicated. LEVEL OF EVIDENCE I, RECOMMENDATION A.
- Laparoscopy is an option for patients who are not candidates for neoadjuvant therapy (in the absence of M1 disease). EVIDENCE LEVEL II, RECOMMENDATION A.
- PET/CT is not recommended in routine practice, but may be used for metastatic disease exclusion when other diagnostic methods do not reveal it. EVIDENCE LEVEL III, RECOMMENDATION C.

UDE is a crucial procedure in the diagnosis, staging, treatment and palliative resection of patients with gastric cancer. The endoscopic examination can identify the location of the neoplasia, allowing biopsy of suspicious lesions [2].

After diagnosis, staging should be carried out by using chest and abdomen CT scans. When clinically indicated, pelvis CT can complement staging. In patients without distant metastases, PET/CT can be carried out in an attempt to increase the accuracy in detecting a distant disease from the original location [2].

If distant metastases are detected, the patient receives palliative treatment. If metastatic disease is absent, the patient is a candidate for surgical treatment, and therefore should undergo echoendoscopy to evaluate the regional lymph nodes and to determine whether or not neoadjuvant treatment may be indicated [2].

Endoscopic ultrasound or echoendoscopy is essential for the initial staging of gastric cancer and can be carried out before surgical treatment to assess the extent of the tumour invasion, the presence of abnormal or enlarged regional lymph nodes and to determine the presence of ascites and metastases in proximal organs [10, 14]. A laparoscopy examination can be conducted before gastrectomy and is independent of the presence or absence of locoregional invasion. Laparoscopy is highly sensitive for the detection of peritoneal metastasis or involvement of the gastric serosa. It may alter the therapeutic decision in up to 20% of the cases considered as M0 in conventional staging tests [25, 32]. Cytology testing of peritoneal fluid during laparoscopy can facilitate staging. If peritoneal washing is positive, the disease is considered as M1 even in the absence of visible distant metastases. Tumour markers have no established role in the diagnosis of gastric tumours.

### Pathology

#### Histopathological classification of gastric tumours. How should it be conducted?

- Histopathological study of gastric tumours should comply with the World Health Organisation’s (WHO) classification. LEVEL OF EVIDENCE I, RECOMMENDATION A.
- The histopathological classification of gastric adenocarcinomas into intestinal or diffuse-type carcinomas should be carried out according to the WHO or Laurén’s classifications. LEVEL OF EVIDENCE I, RECOMMENDATION A.

Gastric adenocarcinoma is one of the few malignant neoplasms in which an infectious agent is recognised to contribute to its aetiology [93]. *Helicobacter pylori* was first isolated in 1982 from a gastric biopsy culture. It promotes an inflammatory response in the gastric mucosa of infected individuals associated with the development of gastritis and peptic ulcers. In 1994, the WHO recognised *H. pylori* infection as a primary cause of gastric adenocarcinoma [51]. The incidence of gastric cancer attributable to *H. pylori* in the population of developing countries contributed to 78% of tumours located below the gastric cardia [93].

The evolution of cases with *H. pylori* infection varies according to individual genetic characteristics and environmental factors. Only a small percentage of infected individuals would develop gastric cancer (three cases per year for every 10,000 infected individuals).

According to the classification of the WHO, published in 2010 [13], gastric cancers are classified into the following:

- NOS adenocarcinoma (without other specifications).
- Papillary adenocarcinoma.
- Tubular adenocarcinoma.
- Mucinous adenocarcinoma;
- Poorly cohesive carcinoma;
- Mixed carcinoma.

Over 90% of gastric cancers are adenocarcinomas.

The terms tubular, papillary and mucinous refer to histological variants of intestinal-type adenocarcinoma. Intestinal tumours do not form tubules and their cells are usually arranged in tightly cohesive aggregates, without polarity or gland formation [13].

When describing the histological subtypes of adenocarcinoma, the tumour should be graded based on cell differentiation [13]. Therefore, the tumour can be classified as:

- Grade I: well differentiated;
- Grade II: moderately differentiated;
- Grade III: poorly differentiated.

The poorly cohesive carcinoma and mixed carcinoma were not included in this classification because these subtypes already have an inherent high grade.

Despite the specific histological variant, the depth of tumour invasion in the gastric wall determines the primary stage of the tumour (T). The TNM classification for gastric cancer staging is independent of the tumour location and correlates with five-year survival rates, which can vary from about 70% for stage IA to 4% for stage IV.

It is recommended that a minimum number of 20 lymph nodes is evaluated by the pathologist to improve the N staging accuracy. It is noteworthy that the evaluation of the largest possible number of lymph nodes achieves the best results.

#### What elements should be described in the pathology report of gastric cancer?

We recommend the description of the following items in the pathology report of gastric cancer:

- RECOMMENDATION A
  - Location
  - Histological type
  - Degree of differentiation
  - Level of tumour invasion (penetration)
  - Presence of angiolymphatic invasion
  - Total number of lymph nodes dissected and number of positive lymph nodes
  - Margins status
  - Staging according to TNM criteria

The histological type and its degree of differentiation are defined according to the WHO’s criterion, as previously mentioned [13]. Tumours involving the gastroesophageal junction (GEJ) that have an epicentre within 2 cm proximal to the gastric cardia or proximal stomach should be classified as oesophageal cancer. Tumours with epicentre located more than 2 cm distal from the GEJ, regardless of its involvement, should be classified as gastric cancer according to TNM parameters [38]. For data reporting purposes, Barrett’s oesophagus showing high-grade dysplasia should be reported as a carcinoma *in situ*.

Regarding the margins, they are segmented into proximal, distal and radial. The radial margin represents the soft tissue margin closest to the deepest penetration of the tumour. The sections for evaluating the margins of the proximal and distal resections can be obtained in two orientations [27, 30] as follows:

1. Facial sections parallel to the margin;
2. Longitudinal sections perpendicular to the margin.

The section choice impacts the definition of margin status. The distance from the tumour margin to the nearest resection margin should be measured if all margins are not involved in invasive carcinoma. The margins of proximal and distal resection should be evaluated for Barrett’s oesophagus and squamous and glandular dysplasia if they are not involved with invasive carcinoma [30].

Tumours resected by endoscopy should be evaluated at the deepest (vertical) margin. The lateral margins can be considered relevant in cases of endoscopic submucosal dissection and when a complete resection sample is available [84]. We recommend the use of a modified Ryan method to determine the efficacy of the therapy [84] (Table 3).

#### How to determine gastric cancer response to neoadjuvant treatment by pathology

- The treatments’ response should consider the percentage of viable neoplastic cells. EVIDENCE LEVEL V, RECOMMENDATION A.

A reliable method to assess the response to treatments considers the percentage of viable cells and the determination of cells that are not classified as a viable cell, such as necrosis, fibrosis and mucus [84]. The 8th Edition of the American Joint Committee on Cancer (AJCC) recommends the use of Ryan’s modified method [84] (Table 2). Besides, the verification of lymph node remission corroborates the treatment response information and provides prognostic relevance, particularly for cases with a more significant response of the primary tumour to treatment [95].

#### When to assess HER2 status, PD-L1 and microsatellite instability (MSI) in gastric cancer?

- The assessment of HER2 status is optional in biopsies of early adenocarcinomas. EVIDENCE LEVEL IV, RECOMMENDATION C.
- The assessment of HER2 status should be carried out in biopsies of metastatic adenocarcinoma. LEVEL OF EVIDENCE I, RECOMMENDATION A.
- The assessment of the HER2 status in metastatic gastric adenocarcinomas can be carried out on surgical specimens, biopsies or cell blocks of primary or metastatic tumour cells, by using immunohistochemistry and interpreted according to the recommended scoring. LEVEL OF EVIDENCE I, RECOMMENDATION A.
- MSI should be assessed on samples of advanced adenocarcinoma. EVIDENCE LEVEL IV, RECOMMENDATION A.
- MSI should be assessed on samples of early gastric adenocarcinoma. EVIDENCE LEVEL IV, RECOMMENDATION B.
- The determination of PD-L1 should be carried out on advanced adenocarcinoma. EVIDENCE LEVEL II, RECOMMENDATION A
- NTRK fusion testing should be assessed on samples of advanced adenocarcinoma, EVIDENCE LEVEL III, RECOMMENDATION A

An increasing number of studies have been reporting the overexpression of HER2 in a subgroup corresponding to 20%–30% of patients with gastric and oesophageal adenocarcinoma. HER2 is a member of the HER family, which encompasses several growth factor receptors, including HER1, HER3 and HER4. They are involved with growth control, proliferation and cell survival [100]. Overexpression of the HER2 protein and its gene amplification are more heterogeneous in gastric cancer than in breast cancer.

The assessment of the HER2 status is critical in defining which patients with advanced disease could benefit from the use of anti-HER2 therapies and evaluation can be carried out on surgical specimens, biopsies and cell blocks (primary or metastatic tumour) [90]. HER2 overexpression in tissue samples should be evaluated with immunohistochemistry (IHC), using Hoffman’s score. Patients with a 2+ score should be further tested with ISH methods [49].

The immediate specimen fixation in buffered formalin during 20–30 minutes is fundamental to histopathological analysis. It is noteworthy that, in gastric and GEJ adenocarcinomas, the positivity of HER2 in the cell membrane found in immunohistochemistry tests is lateral and basal. There is no staining of cellular luminal surface. For diagnosis in surgical specimens, HER2 positivity should be present in more than 10% of the cells. In biopsies, a sample with clustered five positive cells is sufficient for diagnosis [49].

MSI features change in regions with repeated DNA sequences (microsatellites), resulting from mutational inactivation or epigenetic silencing of DNA repair genes (e.g., *MSH1*, *MSH2*, *MSH6* and *MLH1*), being the primary cause of genetic instability in tumours. Tumours with either MSI or Epstein–Barr Virus positivity can stimulate the immune system, promoting susceptibility to immunotherapy with checkpoint inhibitors [81]. However, due to the absence of significant evidences, there is no consensus about the best technique to carry out the MSI test, whether by PCR or immunohistochemistry, since the correlation between both is high and without significant difference [96].

For oesophageal, EGJ and gastric tumours, PD-L1 expression should be determined by using combined positive score (CPS) instead of tumour proportion score (TPS). CPS is the number of PD-L1 staining cells (tumour cells, lymphocytes and macrophages) divided by the total number of viable tumour cells, multiplied by 100. When compared to TPS, CPS is a more sensitive prognostic biomarker in these tumour types [125].

Although generally rare,* NTRK* gene fusions are oncogenic drivers of various adult and paediatric tumours, including oesophageal and EGJ carcinomas. Several methods have been used to detect *NTRK* gene fusions, including immunohistochemistry, FISH, reverse transcriptase polymerase chain reaction (RT-PCR) and DNA- or RNA-based next generation sequencing, as described elsewhere [94].

Next generation sequencing (NGS) can be used in cases of insufficient tissue sample for identification of HER2 overexpression, MSI and NTRK gene fusions. Liquid biopsy is yet not easily available in Brazil.

### Endoscopic treatment

#### When to consider endoscopic resection in gastric cancer?

- Endoscopic mucosal resection and submucosal dissection should be carried out only in reference centres and by professionals with adequate certification to carry out such procedures. EVIDENCE LEVEL V, RECOMMENDATION A.
- Mucosectomy or endoscopic mucosal resection is an appropriate treatment alternative for well-differentiated early gastric tumours (intestinal type) with a diameter of ≤2 cm. LEVEL OF EVIDENCE I, RECOMMENDATION A.
- Mucosectomy or endoscopic mucosal resection is an appropriate treatment alternative for early and well-differentiated gastric tumours (intestinal type) that present as flat or depressed ≤1 cm, without ulceration. LEVEL OF EVIDENCE I, RECOMMENDATION A.
- In order for endoscopic resection to be carried out, the tumour should not infiltrate the deep submucosa or invade lymphovascular vessels and regional lymph nodes. LEVEL OF EVIDENCE I, RECOMMENDATION A.
- Gastric echoendoscopy is optional and may be carried out before endoscopic mucosal resection to assess the depth of tumour invasion. EVIDENCE LEVEL V, RECOMMENDATION C.

Early gastric cancer is defined as a neoplasm that is limited to the mucosa or submucosa. The curative treatment of the early disease depends on the complete removal of the tumour and regional lymph nodes. Fortunately, most early gastric tumours do not present lymph node metastasis. Lymphatic involvement, tumour size and depth of submucosal invasion are prognostic factors [6], making the curative treatment of these lesions possible by endoscopy [24].

Endoscopic resection includes endoscopic mucosal resection (EMR) or mucosectomy, involving resection with metal clips and submucosal dissection (SMD), which involves several endoscopic tools to dissect or detach submucosal lesions. Thus, lesions limited to the mucosa and superficial layers of the submucosa seem to be the most appropriate for curative endoscopic treatment.

EMR is the most well-known resection technique worldwide. The most exceptional experience with different endoscopic resection techniques is from Japan. The Japanese Society of Gastrointestinal Endoscopy (JSGE) elaborated a classification system for early gastric cancer based on the findings of endoscopy and echoendoscopy. This system recognises the following four types of early endoluminal tumours:

- Type I – polypoid or protruding lesions
  - Ip – pedunculated
  - Isp – subpedunculated
  - Is – sessile
- Type II – flat lesions
  - IIa – surface elevation
  - IIb – flat
  - IIc – flat and depressed
  - IIc + IIa – an elevated area in a depressed lesion
  - IIa + IIc – a depressed area in an elevated lesion
- Type III – ulcerated lesions
- Type IV – lesions with a lateral invasion

According to JSGE, the accepted indications for EMR, are: elevated and well-differentiated tumours, ≤2 cm in diameter [28]; and depressed lesions ≤1 cm. These lesions should also be well differentiated (intestinal adenocarcinoma), limited to the mucosa and without lymphatic or vascular invasion [101, 123]. Regardless of tumour size, undifferentiated tumours should be removed surgically. The risk of lymph node metastasis seems to depend more on the presence of an ulcer than on the depth of the early tumour invasion. Therefore, an endoscopic approach is not indicated in those cases [56].

SMD is a new alternative endoscopic treatment technique that offers endoscopists the possibility to remove whole mucosal and submucosal lesions *en bloc* using an endoscopic scalpel with a porcelain tip [45, 46, 87, 124]. The removal of early gastric cancer is the most successful experiment using this technique, although it is also applicable to oesophageal and colon lesions [19, 45, 46, 55, 67, 87, 89].

In a meta-analysis of 15 non-randomised studies, SMD was compared with EMR in lesions of the oesophagus, stomach and colon. A subgroup analysis showed better rates for curative resection and local recurrence when using SMD. The best performance, however, was followed by a higher frequency of bleeding and perforation [21].

A second meta-analysis recently published by Park *et al* [92] compared SMD with EMR in cohort studies that involved only early gastric cancer. The results showed that SMD is significantly superior to REM for *en bloc* resection, complete resection, curative resection and local recurrence rate. Although the risk of perforation was higher with SMD (HR = 0.65; CI = 95% 0.80–5.38), the risk of bleeding did not differ between the two procedures (HR = 1.22; CI = 95% 0.76–1.98). These results led investigators to believe that, in skilled and experienced hands, the benefit of SMD still outweighs its risks [92]. More data on the potential impact of the two techniques on long-term survival are still needed to strengthen the role of SMD.

Echoendoscopy is a sensitive method that determines the depth of the tumour invasion in the stomach wall in patients with gastric cancer. However, the role of echoendoscopy in identifying ideal candidates with early gastric cancer for endoscopic resection is still debatable.

In general, endoscopic resection is an option for curative treatment of early gastric cancer with potential chances of long-term cure. However, because of the variety of related complications, it should be carried out in referral centres and by professionals with adequate certification to carry out such procedures.

### Non-metastatic disease—gastric cancer surgery

#### In what situations should surgery be considered in patients with gastric cancer?

- All cases of gastric cancer should be discussed in a multidisciplinary board. EVIDENCE LEVEL II, RECOMMENDATION A.
- Patients with gastric cancer with the absence of distant metastases should be considered for surgery with curative intent unless candidates present criteria for endoscopic resection. EVIDENCE LEVEL III, RECOMMENDATION A.

The curative treatment of gastric adenocarcinoma is complex and may involve variations of therapeutic modalities that include surgery, chemotherapy and radiotherapy. Thus, we recommend a multidisciplinary discussion of all cases of gastric adenocarcinoma to establish an appropriate therapeutic strategy for each patient. In the absence of locoregional involvement and distant metastases, the patient should be referred for surgery. When regional disease is detected in a patient without distant metastasis, neoadjuvant therapy should be considered. Patients with confirmed metastatic disease should be treated with systemic chemotherapy or supportive care [2].

Surgical resection provides the best chances of long-term survival for patients with localised gastric cancer, particularly if combined with adjuvant chemotherapy, perioperative chemotherapy or postoperative chemoradiotherapy [7, 27, 72]. A proper therapy begins with an accurate staging of the extent of the disease. Patients without distant metastases are potential candidates for surgical treatment, aiming to the removal of the primary lesion with clear longitudinal and circumferential margins [88].

#### What is the appropriate extent of gastric resection?

- Total gastrectomy should be carried out in patients with diffuse infiltrative gastric tumours. LEVEL OF EVIDENCE I, RECOMMENDATION A.
- Partial gastrectomy should be carried out in patients with a distal stomach tumour. LEVEL OF EVIDENCE I, RECOMMENDATION A.
- For gastrectomy, the route through abdominal access is preferable. EVIDENCE LEVEL II, RECOMMENDATION B.
- Prophylactic removal of the spleen or pancreas is not recommended when carrying out gastrectomy. LEVEL OF EVIDENCE I, RECOMMENDATION A.
- Partial gastrectomy should be carried out on antrum tumours, with adequate margins. LEVEL OF EVIDENCE I, RECOMMENDATION A.
- Partial gastrectomy can be carried out in cases of tumours of the mid-third body. EVIDENCE LEVEL IV, RECOMMENDATION B.
- In the case of tumours with oesophagus invasion, the objective is to obtain negative margins. EVIDENCE LEVEL IV, RECOMMENDATION A.

The resection extent is determined primarily by the location of the tumour. Partial gastrectomy is preferable as long as the margin is adequate, and the tumour is not a diffuse infiltrative type [17, 31]. The recommended margin resection extension is 3 cm for well-circumscribed tumours and Borrmann type I or II, and at least 5 cm for other tumours [101]. It is crucial to consider the analysis of the margins during the operation.

For tumours close to GEJ, total gastrectomy with resection of about 5 cm from the distal oesophagus is the recommended operation. The route access through laparotomy has fewer postoperative complications compared to the thoracoabdominal approach [102]. Prophylactic removal of the spleen and pancreas is not recommended. It is indicated only in cases of tumour invasion [127].

#### What is the appropriate lymphadenectomy extent in the management of gastric cancer?

- Gastrectomy with D2 lymphadenectomy is the surgery of choice for the surgical treatment of stage I, II and III gastric cancer, except for cases eligible for endoscopic resection. EVIDENCE LEVEL I, RECOMMENDATION A.
- D2 lymphadenectomy should be carried out in centres of high expertise by an experienced surgical team. EVIDENCE LEVEL I, RECOMMENDATION A.

The extension of lymphadenectomy is one of the most complex and controversial topics in the management of gastric cancer. The lymphatic drainage of the stomach has been carefully studied by Japanese surgeons and divided into 16 stations: stations 1 to 6 are perigastric and the remaining 10 are located proximal to large vessels, behind the pancreas and along the aorta [101]. From the location of the lymph node groups, the extension of the lymphadenectomy can be classified into:

- D1 lymphadenectomy: Dissection limited to perigastric lymph nodes.
- D2 lymphadenectomy: More extensive lymph node dissection, which includes the removal of lymph nodes along with the hepatic, left gastric, celiac and splenic arteries, in addition to the lymph nodes in the splenic hilum (stations 1–11).
- D3 lymphadenectomy: Overextended dissection is used by many specialists to designate a D2 lymphadenectomy associated with the removal of the hepatic portal and along with the aorta lymph nodes (stations 1–16).

The arguments favouring the extended lymphadenectomy (D2 or D3 versus D1) are reasonable. Removing a significant number of lymph nodes may result in better staging, while reducing the risk of residual disease. However, criticisms point out increased surgery-related morbidity and mortality and the non-significant survival benefit in most large randomised trials [11, 29, 34].

The number of compromised lymph nodes is a major parameter in determining survival associated with disease staging [73]. The most widely used gastric cancer staging system, UICC/AJCC, recommends examining at least 15 lymph nodes to define the N stage [37]. Despite the absence of scientific evidence since the first version of the JGCA published in 1962, the extended lymphadenectomy technique was recommended for the treatment of all gastric tumours in Japan. Numerous retrospective studies have demonstrated the improvement in the survival of patients undergoing D2 compared to conservative procedures [62, 75, 85, 99]. In the 1990s, randomised trials were designed to elucidate the superiority of D2 gastrectomy. In Europe, 400 patients in stages I to III were randomised to gastrectomy with D1 or D2 dissection. No significant difference in five-year survival was observed, and the morbidity and mortality in patients undergoing D2 were significantly higher than in those undergoing D1 dissection.

From 1989 to 1993, a large clinical trial with 711 participants was carried out in the Netherlands, aiming to determine the best treatment [12]. The initial results demonstrated that the morbidity and mortality in patients undergoing D2 were significantly higher than those undergoing D1 dissection. The authors concluded that D2 lymph node dissection should not be indicated in the treatment of gastric cancer. However, concerns were raised regarding a large number of surgeons and hospitals, which were unlikely to carry out standardised procedures. The absence of lymph nodes was observed in more than 50% of D2 lymphadenectomies, while excess lymph nodes were found in 42% of D1 lymphadenectomies, and the high mortality rate of patients submitted to D2 lymphadenectomy was problematic [18]. Nevertheless, after 15 years of follow-up, the authors confirmed that lymphadenectomy D2 was associated with a lower incidence of locoregional recurrences and fewer deaths than D1 lymphadenectomy. By updating the initial conclusions, the authors considered recommending D2 lymphadenectomy as initial therapy for gastric adenocarcinoma [111]. In Asia, a randomised study of five-year survival assessment showed a significantly superior OS in patients undergoing D2 lymphadenectomy [122]. A study conducted in Japan considered it unnecessary to expand D2 dissection with resection of paraaortic lymph nodes [103]. In summary, retrospective studies suggested improved survival in patients undergoing D2 lymphadenectomy. However, these results are difficult to confirm in prospective studies. The D2 lymphadenectomy provides adequate staging, increases survival in some subgroups of patients and does not increase complications and mortality when carried out by experienced surgeons.

Although data derived from the Western investigations are less robust than those from the Eastern investigations, we recommend gastrectomy with D2 lymphadenectomy without splenectomy. And of course, the number of lymph nodes dissected is directly related to the lymph node extension.

#### Is the laparoscopic approach an appropriate alternative for gastrectomies?

- Gastrectomy with D2 lymphadenectomy can be carried out with laparoscopy. EVIDENCE LEVEL II, RECOMMENDATION B.
- Partial gastrectomy by laparoscopy is feasible in early tumours, with similar postoperative morbidity and mortality to open surgery. LEVEL OF EVIDENCE I, RECOMMENDATION A.
- Laparoscopic gastrectomy and open surgery have similar survival rates. EVIDENCE LEVEL III RECOMMENDATION B.

Laparoscopy-assisted gastrectomy has some advantages over open gastrectomies, such as less bleeding and inflammation, better lung function and milder pain in the postoperative period, shorter time to re-establish food intake and start flatus and shorter hospitalisation [61].

Considering the complexity in using the technique, which involves blood supply, stomach lymphatic drainage and difficulties inherent to a surgical process, laparoscopic-assisted gastrectomy is recognised as a procedure requiring a proper learning curve [59]. Laparoscopy is used in many other surgeries unrelated to the stomach, such as cholecystectomies, urological procedures and colorectal surgeries, all equally dependent on the surgeon’s experience. Therefore, depending on the background, which may vary from 8 to 20 operations, and considering the complexity of each case, laparoscopy has lower complication rates [80, 106, 108].

Approximately 50 operations may be necessary to achieve optimal proficiency for laparoscopic-assisted gastrectomy [59]. The number of surgeries needed would vary substantially according to some factors, such as the extent of lymphadenectomy, additional procedures and patients with incorrect staging plus surgeon’s personal characteristics [22].

#### When is laparoscopy recommended for staging during the management of gastric cancer patients?

- Curative gastrectomy should be initiated by laparoscopy to identify potential intracavitary tumour implants. EVIDENCE LEVEL II, RECOMMENDATION A.
- Candidates for neoadjuvant treatment should previously undergo laparoscopic staging with peritoneal lavage. EVIDENCE LEVEL II, RECOMMENDATION A.
- Initiate upfront surgery with laparoscopy, if accessible, for patients with cT3, cT4 or cN+ disease (there was no consensus about the procedure in earlier disease). EVIDENCE LEVEL III, RECOMMENDATION A.
- For unconfirmed peritoneal involvement found on CT scan, laparoscopy should be carried out with peritoneal lavage for diagnostic confirmation purposes. EVIDENCE LEVEL III, RECOMMENDATION A.

Because of the high sensitivity in identifying tumour implants, we recommend starting a curative surgical procedure with laparoscopy assistance. Visualisation of the peritoneal cavity by laparoscopy allows the identification of small volume tumour implants and local lymph nodes that were not identified in the preoperative radiological examinations. In fact, by using laparoscopy, peritoneal metastases can be documented in up to 30% of patients with gastric cancer with negative abdominal tomography, which would be considered potentially curable by surgery [32]. Besides, laparoscopy allows peritoneal lavage for cytology study. In the case of neoplastic cell detection, the M1 disease is confirmed and a poor prognosis may be expected. Patients with advanced tumours (T3 or T4) should be considered for staging with laparoscopy with lavage [76].

### Non-metastatic disease—chemotherapy and radiotherapy in gastric cancer

#### When should radiotherapy be considered in the neoadjuvant scenario?

- Radiotherapy in the neoadjuvant setting is an acceptable therapeutic option in selected cases of gastric cancer. EVIDENCE LEVEL III, RECOMMENDATION C.

Radiotherapy has a role in the curative treatment of gastric cancer. This therapeutic modality improves the local disease control, reducing recurrence while increasing the chances of cure for these patients. Radiotherapy can be carried out before or after surgical resection (neoadjuvant or adjuvant). Currently, no phase III clinical trials support the role of neoadjuvant radiotherapy in the treatment of gastric cancer.

#### In what situation should neoadjuvant chemotherapy (perioperative) be considered in patients with gastric cancer?

- Neoadjuvant or perioperative chemotherapy is the treatment of choice for patients with locally advanced gastric cancer, particularly in clinical stages T2, T3, T4 or N+, considering adequate clinical staging. LEVEL OF EVIDENCE I, RECOMMENDATION A.
- For patients with good *performance status*, the preferred regimen is FLOT [docetaxel, oxaliplatin, leucovorin and 5-fluorouracil (5-FU)] for neoadjuvant or perioperative chemotherapy. LEVEL OF EVIDENCE I, RECOMMENDATION A.
- For the Eastern Cooperative Oncology Group (ECOG)-2 patients, the preferential regimen includes a doublet (fluoropyrimidine/platinum) for neoadjuvant or perioperative chemotherapy as these patients may not be fit enough for a triplet regimen because of age, comorbidities and/or clinical contraindications. LEVEL OF EVIDENCE I, RECOMMENDATION A.
- When a doublet regime is considered, the FOLFOX (leucovorin, 5-FU and oxaliplatin) scheme is the preferred combination. EVIDENCE LEVEL III RECOMMENDATION A.
- We do not recommend epirubicin. EVIDENCE LEVEL II, RECOMMENDATION A.

Contrasting to the United States, the standard treatment for gastric cancer in many European countries combines neoadjuvant or perioperative chemotherapy. In addition to the early control of micrometastases, neoadjuvant chemotherapy promotes tumour reduction that may facilitate surgery, increase patient adherence to systemic treatment and test the neoplasias’ sensitivity to chemotherapy.

Three large clinical trials compared surgery with and without neoadjuvant or perioperative chemotherapy, and two of them demonstrated survival benefits using the therapeutic strategy [27, 105, 126].

The multicentre MAGIC study investigated the efficacy of pre- and post-operative chemotherapy in patients with gastric (*n* = 372 patients) and GEJ adenocarcinomas (*n* = 131 patients). The regimen consisted of three cycles of epirubicin, cisplatin and 5-FU before surgery and three cycles after surgery. Unfortunately, only 42% of patients in the chemotherapy group completed the study according to the protocol. Approximately 34% of patients completed all preoperative chemotherapy cycles and underwent surgery, but did not receive postoperative chemotherapy. Despite this, surgical treatment combined with chemotherapy resulted in a significant benefit of OS (HR for death = 0.75; 95% CI 0.60–0.93; *p* = 0.009) and DFS (HR for progression = 0.66). The authors also described a 36% 5-year survival rate, compared to 23% in the surgery-only group, in favour of the perioperative chemotherapy group (3).

The French multicentre clinical trial ACCORD07-FFCD was comparable to the MAGIC study [126]. In this study, 224 patients with gastric (25%), cardia (64%) and distal oesophagus adenocarcinomas (11%) were randomised to surgery alone or to receive perioperative chemotherapy and surgery. Chemotherapy consisted of two to three cycles of cisplatin and 5-FU before surgery, and three to four cycles after surgery. No difference was observed in surgery-related morbidity or mortality between groups. Complete R0 resections were more frequent in patients treated with preoperative chemotherapy than in patients who underwent surgery alone (87% versus 74%, *p* = 0.04). After a 5-year follow-up period, the use of perioperative chemotherapy was associated with a 35% reduction in disease recurrence risk (34% five-year DFS versus 19%, *p* = 0.003) and a 31% decrease in the risk of death (5-year OS of 38% versus 24%, *p* = 0.02).

Unlike the previously mentioned studies, the EORTC 40954 did not demonstrate a survival benefit in patients with locally advanced gastric and GEJ cancer who received neoadjuvant chemotherapy [105]. Patients were randomised to either surgery alone or neoadjuvant chemotherapy, followed by surgery. The neoadjuvant chemotherapy regimen consisted of two cycles of 48 days with cisplatin D1/15/29, followed by 5-FU in bolus and infusion of 5-FU for 24 hours. The study was interrupted because of low recruitment when only 114 of the 360 planned participants had been accrued. The percentage of R0 surgeries was higher in the experimental group (81.9% versus 66.7%; *p* = 0.036), at the expense of a non-significant increase in the rate of postoperative complications (27.1% versus 16.2%; *p* = 0.09). After a 4.4-year follow-up, the study showed no benefit in terms of OS (HR for death = 0.84; 95% CI 0.052–1.35; *p* = 0.446).

A meta-analysis published by a Chinese group selected all clinical trials involving preoperative systemic treatment for advanced gastric cancer. A total of 2,271 patients included in 14 studies were divided in groups that received neoadjuvant therapy (*n* = 1,054) and the control group (*n* = 1,217). Neoadjuvant chemotherapy significantly improved the survival rate (HR = 1.27; 95% CI 1.04–1.55) and the rate of complete resections—R0 (OR = 1.51; 95% CI 1.19–1.91) [68].

The neoadjuvant chemotherapy in the treatment of advanced gastric cancer has been increasingly accepted by specialists. The FLOT4 trial [3] was a multicentre, randomised phase III study comparing anthracycline-based triplet therapy [epirubicin, cisplatin and 5-FU (ECF) or epirubicin, cisplatin and capecitabine (ECX)] with docetaxel-based triplet therapy [5-FU and leucovorin (LV), oxaliplatin and docetaxel] in the context of perioperative treatment in patients with operable gastroesophageal cancer with 716 patients (360 for ECF/ECX and 356 for FLOT). Three preoperative cycles and three postoperative cycles of 3 weeks of ECF/ECX were compared to four preoperative cycles and four postoperative cycles of FLOT of 2 weeks. Ninety-one percent and 37% of ECF/ECX patients completed the pre- and post-operative cycles, respectively, compared to 90% and 50% with FLOT.

The median follow-up was 43 months, with 369 patients deceased (203 in the ECF/ECX group and 166 in the FLOT group). Patients in the FLOT group showed superior OS (median OS: 35 months for ECF/ECX versus 50 months for FLOT; HR: 0.77 (0.63−0.94); *p* = 0.012), superior OS in 3 years (48% in the ECF/ECX group versus 57% in the FLOT group) and superior PFS (median: 18 months in the ECF/ECX group versus 30 months in the FLOT group; HR: 0.75 (0.62–0.91); *p* = 0.004).

The groups were similar in terms of perioperative complications (50% in the ECF/ECX group versus 51% in the FLOT group). Mortality at 30 and 90 days was 3% and 8% in the ECF/ECX group and 2% and 5% in the FLOT group, respectively. Grade 3 and 4 adverse events were distinct, with more patients with nausea and vomiting with ECF/ECX and more neutropenia with FLOT.

#### In what situation should adjuvant chemotherapy be considered in patients with gastric cancer?

- Adjuvant chemotherapy alone is recommended for patients with stage II–III gastric adenocarcinoma undergoing D2 resection. LEVEL OF EVIDENCE I, RECOMMENDATION A.
- Platinum and fluoropyrimidine-based regimen is recommended. LEVEL OF EVIDENCE I, RECOMMENDATION A.
- Adjuvant chemotherapy alone should preferably be carried out with a combined regimen of capecitabine and oxaliplatin (CAPOX). If not possible, a combination of regimens with a platinum agent and a fluoropyrimidine is recommended. LEVEL OF EVIDENCE I, RECOMMENDATION A.

The scientific debate on the adjuvant treatment of gastric adenocarcinoma is very dynamic, with a large number of clinical trials and studies available in the medical literature. The proposed adjuvant chemotherapy regimens vary between studies that contain or not anthracycline [5, 7, 16, 26, 30, 33, 40, 50, 63, 69, 82, 83].

Because of the variability in results of the available studies, several meta-analyses were carried out to clarify the impact of adjuvant chemotherapy on the survival of patients with gastric cancer [70, 74, 90, 91, 113]. Among these studies, the most recent report analysed 3,838 patients included in 17 randomised clinical trials, who were followed-up for more than 7 years [91]. The role of each chemotherapy regimen, including monotherapy, combined therapy with or without anthracyclines, and other regimens were also assessed. Adjuvant chemotherapy was associated with a significant benefit of OS (HR = 0.82; 95% CI 0.76–0.90; *p* < 0.001) and disease-free survival (HR = 0.82; CI 95 % 0.75–0.90; *p* <0.001). There was no significant difference in survival rates according to chemotherapy regimens.

The benefit of adjuvant therapy using capecitabine combined with oxaliplatin was assessed in the CLASSIC clinical trial [7]. This Korean study randomised a total of 1,035 patients with stage II–IIIB gastric adenocarcinoma who underwent D2 resection, followed by eight cycles of adjuvant CAPOX (*n* = 520 patients) or for surgery alone (n = 515 patients). The results showed a 3-year DFS rate of 74% in the experimental group versus 59% in the control group (HR = 0.56; 95% CI 0.44–0.72; *p* < 0.0001) and a non-significant gain in OS (83% versus 78%, HR for death = 0.72; 95% CI 0.52–1.00). As expected, Grade 3 and 4 toxicity increased from 6% in the control group to 56% in the experimental group. The CLASSIC trial strengthened the role of adjuvant treatment in advanced gastric cancer and consolidated the activity of the combined fluoropyrimidine and oxaliplatin regimen in this population, offering options for adjuvant treatment (when the patient is referred to an oncologist after surgery), especially for patients who are not candidates for concomitant radiotherapy.

#### In what situation should adjuvant chemotherapy–radiotherapy (CT–RT) be considered in patients with gastric cancer?

- Clipping the surgical bed during surgery is recommended in patients who are candidates for adjuvant radiotherapy to optimise the planning of the irradiation field. EVIDENCE LEVEL V, RECOMMENDATION A.
- Radiotherapy should be added to the 5-FU/LV adjuvant treatment in patients with gastric or GEJ adenocarcinoma T3 stage with positive lymph nodes or T4 submitted to D0 or D1 resection. LEVEL OF EVIDENCE I, RECOMMENDATION A.
- Radiotherapy can be added to 5-FU/LV-based adjuvant treatment in patients with gastric adenocarcinoma or GEJ T3 stage with positive lymph nodes or T4 submitted to D2 resection. EVIDENCE LEVEL II, RECOMMENDATION C.
- The addition of chemotherapy, other than 5-FU/LV, is not recommended for patients on the adjuvant CT–RT schedule. EVIDENCE LEVEL II, RECOMMENDATION C.
- Patients with gastric or GEJ adenocarcinoma and R1/R2 resection should receive adjuvant CT–RT. EVIDENCE LEVEL V, RECOMMENDATION A.
- There is no evidence of adjuvant CT–RT benefits in patients with gastric or GEJ adenocarcinoma who received perioperative chemotherapy and achieved R0 resection. EVIDENCE LEVEL V, RECOMMENDATION A.

Three randomised clinical trials demonstrated a significant survival benefit with the postoperative addition of CT–RT compared to surgical resection alone. The US study INT 0116, published by Macdonald *et al* [72], allocated patients who underwent curative gastric cancer surgery for observation or to receive adjuvant treatment with CT–RT. The treatment regimen consisted of the first cycle of CT using 5-FU 425 mg/m²/day and LV 20 mg/m²/day, followed by CT–RT, which started 4 weeks after the beginning of the first CT cycle. CT–RT consisted of a total accumulated radiation dose of 45 Gy, divided into 1.8 Gy/day, 5 days a week, associated with a reduced dose of 5-FU (400 mg/m²) and LV in the first and last 4 or 3 days of radiotherapy. Four weeks after the end of radiotherapy, two additional five-day cycles using 5-FU (425 mg/m²) and LV were administered with an interval of 4 weeks between them. After 3 years, this study demonstrated a 9% survival rate for patients treated with CT–RT (50% versus 41%, *p* = 0.005). The adjuvant therapy decreased the percentage of tumour recurrence from 64% to 43%, and median recurrence-free survival increased from 19 to 30 months [72]. After more than 10 years of follow-up, the results showed a strong benefit of postoperative CT–RT. The HR for OS was 1.32 (95% CI 1.10–1.60; *p* = 0.0046) and the HR for PFS was 1.51 (95% CI 1.25–1.83; *p* < 0.001). Adjuvant CT–RT provided a substantial reduction in general and locoregional recurrence, becoming a reasonable alternative for surgical resection of gastric cancer [110].

The criticism of this study argues that only 10% of the included patients underwent D2 lymphadenectomy. Therefore, the study’s conclusion does not apply to patients treated with current resection techniques. Despite this, the INT 0116 protocol has become the standard treatment in the United States and is still used in some Brazilian centres.

The US CALGB 80101 clinical trials compared the regimen proposed in INT 0116 versus systemic therapy using ECF before and after 5-FU concomitant with radiotherapy. This study intended to prove whether or not intense chemotherapy associated with radiotherapy would deliver better chances for the patients’ cure. Contrary to initial expectations, the study was negative for its primary and secondary objectives. In a preliminary report, after 3 years of follow-up, OS was not significantly better with ECF in comparison to the regimen with 5-FU alone (52% versus 50%) [43]. Therefore, when considering adjuvant CT–RT, the protocol proposed in the INT 0116 study should be used.

Additional evidence in favour of adjuvant CT–RT is provided by the ARTIST trial, which compared adjuvant CT–RT with adjuvant chemotherapy in 458 patients undergoing complete gastric cancer resection and D2 lymphadenectomy. The control group received six cycles of adjuvant chemotherapy with capecitabine and cisplatin without radiotherapy. The experimental group received adjuvant treatment with two cycles of capecitabine and cisplatin, followed by radiotherapy concomitant with capecitabine followed by two additional cycles of capecitabine and cisplatin. Although the DFS was similar in both arms of the study, in a subgroup analysis, patients with compromised lymph nodes had better outcomes with CT–RT, favouring the experimental arm (77.5% PFS in 3 years versus 72, 3%, *p* = 0.0365) [65].

Therefore, postoperative chemoradiation effectiveness depends on the extent of lymph nodes dissection. Postoperative chemoradiation failed to offer clinical benefit in terms of increased survival in patients who underwent D2 resection while reducing recurrence rates in patients with D0 or D1 resections.

#### What type of adjuvant radiotherapy is recommended for patients with gastric cancer?

- If available, modulated intensity or conformational radiotherapy with 3D planning is recommended. EVIDENCE LEVEL III, RECOMMENDATION A.
- The recommended dose for radiotherapy is 45 Gy, with conventional fractionation of 180 cG in 25 fractions. LEVEL OF EVIDENCE I, RECOMMENDATION A.

When radiotherapy is considered, it is crucial to take into account the technique and the disease volume planned for treatment. Generally, to adequately cover the entire bed of surgical resection and regions with potential micrometastases, large radiotherapy fields are required, with geometrically complexity and proximity to the liver, kidneys and spinal cord. Considerable toxicities may occur in large treatment volumes, especially when concomitant with chemotherapy. In the INT 0116 trial, grade III and IV toxicity rates reached 41% and 32%, respectively [72].

The standard radiation dose of 45 Gy exceeds the tolerance of some healthy tissues that are considered critical, such as liver and kidneys. With conventional 2D and 3D radiotherapy techniques, the coverage of the target field for treatment may be balanced with the preservation of the kidneys.

The introduction of intensity-modulated radiotherapy (IMRT) led radiotherapists to plan larger and better-distributed dose gradients, through geometrically complex volumetric models, allowing the application of high radiation doses with high precision. Several studies have demonstrated the potential of IMRT to reduce the radiation dose in critical healthy structures, but the clinical results are still limited [66, 77, 98, 121].

To date, no large randomised controlled trials comparing IMRT with three-dimensional conformational radiotherapy (3D RDT) are available. A small randomised trial with 57 patients compared clinical outcomes and toxicity with the postoperative CT–RT treatment for gastric cancer using 3D RDT versus IMRT. After 2 years of follow-up, survival rates with 3D RDT and IMRT were 51% and 65%, respectively (*p* = 0.5). The number of local therapeutic failures was also similar between groups. Similar levels of toxicity between groups were observed even though the majority of patients of IMRT received higher doses of radiation [78].

Despite the encouraging results, prospective clinical trials and functional investigations of vital organs, it is still necessary to assess the role of modern radiotherapy techniques in the treatment of gastric cancer.

### Metastatic disease—gastric cancer

#### What regimens and chemotherapy drugs are recommended for the first-line treatment of advanced gastric cancer?

- In the first-line treatment for advanced gastric cancer, a fluoropyrimidine (capecitabine or 5-FU) and a platinum agent (oxaliplatin or cisplatin), or the FOLFIRI (irinotecan, leucovorin and 5-FU) regimens are recommended. LEVEL OF EVIDENCE I, RECOMMENDATION A.
- For young patients with good *performance status* and high volume of disease, chemotherapy with a FLOT or modified DCF (docetaxel, cisplatin and 5-FU) regimens may be used in the first line of treatment for advanced gastric cancer. EVIDENCE LEVEL II, RECOMMENDATION B.
- The modified DCF regimen should preferably be used instead of traditional DCF in the treatment of first-line gastric cancer when using triplet therapy. EVIDENCE LEVEL II, RECOMMENDATION A.
- When the treatment option is chemotherapy regimen without a platinum agent, a combination of irinotecan and 5-FU is recommended. EVIDENCE LEVEL I, RECOMMENDATION A.
- The dual chemotherapy regimen that combines irinotecan and cisplatin may be an alternative in the treatment of advanced gastric cancer. EVIDENCE LEVEL III, RECOMMENDATION B.
- Capecitabine can replace 5-FU in first-line treatment regimens for gastric cancer. LEVEL OF EVIDENCE I, RECOMMENDATION A.
- Oxaliplatin can replace cisplatin in first-line treatment regimens for metastatic gastric cancer. LEVEL OF EVIDENCE I, RECOMMENDATION A.

Many active drugs became available for the treatment of advanced gastric cancer. In the past, cisplatin, methotrexate, 5-FU, mitomycin-C, etoposide, anthracycline and methotrexate-based regimens were prescribed for advanced gastric cancer treatment [100, 104]. Currently, potent agents, such as docetaxel, paclitaxel, capecitabine, oxaliplatin and irinotecan, are available with proven efficacy in first-line therapy.

Combined chemotherapy regimens have shown significant improvement in the outcomes of patients with advanced gastric cancer. In a Cochrane review, the HR for OS in patients treated with combined chemotherapy was 0.85, although the magnitude of benefit was only 1 month when compared to monotherapy (median survival of 7.0 months versus 5.9 months) [118]. The OS of patients treated with modern triple combinations of chemotherapy (DCF, ECF) may reach 9 months [117, 120].

The introduction of the ECF regimen can be considered as a milestone in the evolution of the treatment of metastatic gastric cancer, with significant superior results in comparison to the best regimen available. In a randomised phase III trial, the ECF scheme showed a significant gain of OS of 3.2 months compared to FAMTX (8.9 versus 5.7 months, *p* = 0.009) [120].

The pivotal REAL-2 study compared the ECF regimen to EOF (epirubicin, oxaliplatin and 5-FU), ECX (epirubicin, cisplatin and capecitabine) or EOX (epirubicin, oxaliplatin and capecitabine) (31). This factorial study of non-inferiority with four arms was designed to investigate the possibility of oxaliplatin in replacing cisplatin or capecitabine and in replacing the infusional 5-FU in the ECF regimen, which was proven through an HR for death = 0.86 (95% CI 0.8–0.99) in the capecitabine group, and 0.92 (95% CI 0.8–1.1) in the oxaliplatin group. The median of OS in each group was equivalent: 9.9 months with ECF, 9.9 months with ECX, 9.3 months with EOF and 11.2 months with EOX. Response rates and toxicities were not significantly different between groups. Although the EOX group showed a slightly higher OS, this study does not allow us to conclude that this scheme is superior.

In the phase III trial (TAX 325), 445 patients were randomised to receive either the DCF regimen or CF (cisplatin and fluorouracil). Treatment with DCF resulted in a significantly higher response rate (37% versus 25%), longer time to progression (5.6 versus 3.7 months) and a slight gain in OS (9.2 versus 8.6 months; HR = 1.29; 95% CI 1–1, 6; *p* = 0.02). This trial provided evidence to the approval of docetaxel in the treatment of advanced gastric cancer in different parts of the world. However, the DCF regimen showed an unfavourable toxicity profile with high levels of neutropenia and neutropenic fever [117].

To improve the toxicity profile, the Memorial Sloan Kettering Cancer Centres’ investigators conducted a phase II trial to test DCF modifications. The modified DCF regimen consisted of docetaxel 40 mg/m² in D1, De Gramont’s scheme for 48 hours (D1: 5-FU 400 mg/m², IV, in bolus, followed by 5-FU 2,000 mg/m², IV, in continuous infusion for 48 hours), followed by cisplatin 40 mg/m² in D3. The median OS reported with the modified DCF was 15.1 months [107]. Although no randomised clinical trials have compared the DCF regimen with the modified DCF, this regimen is favourable over the original DCF because of its better safety profile.

The combination of irinotecan with cisplatin was studied in a small phase II trial, which demonstrated a response rate of 58% and a median OS of 9 months [1]. Despite not being studied in phase III trials, the cisplatin and irinotecan regimen (in bolus D1/D8 every 21 days) is considered convenient, safe and effective, being a good therapeutic option to optimise available resources [52].

A French phase II study demonstrated the superiority of the FOLFIRI regimen, initially developed for the treatment of colon cancer with 5-FU/LV associated or not with cisplatin [15]. A more recent phase III study compared ECX and FOLFIRI regimens as first-line treatment for patients with advanced gastric or GEJ adenocarcinoma. The FOLFIRI regimen showed longer time to progression in comparison to ECX (5.1 months versus 4.2 months; *p* = 0.008), but no significant difference in PFS (5.3 months versus 5; 8 months; *p* = 0.96), median OS (9.5 months versus 9.7 months; *p* = 0.95) or response rate (39.2% versus 37.8%). In terms of tolerability, FOLFIRI proved to be superior, presenting a global toxicity profile for grades III and IV of 69% against 84% of ECX (*p* < 0.001), respectively, with lower haematological adverse events (38% versus 64.5% for ECX) [47].

In addition to the REAL-2 study, another phase III trial evaluated oxaliplatin in the treatment of gastric cancer, by comparing the FLO regimen (infusional 5-FU in 24 hours; leucovorin and oxaliplatin every 2 weeks) with the FLP regimen (infusional 5-FU in 24 hours; leucovorin and cisplatin) [4]. PFS and OS were superior in the FLO group (5.8 months versus 3.9 months and 10.7 versus 8.8 months, respectively), although without statistical significance. For the population older than 65 years, the FLO scheme showed a significant gain in OS (13.9 months versus 7.2 months), PFS (6.0 months versus 3.1 months; *p* = 0.029) and response rate (41.3% versus 16.7%; *p* = 0.012). This study endorses the biological rationale for using the FOLFOX regimen in patients with gastric adenocarcinoma, especially in elderly patients.

Considering several options, the value of personalising treatment according to the patients’ profile, available resources and the experience of the medical team is crucial.

#### What regimens and chemotherapy drugs are recommended for the treatment of first-line HER2-positive advanced gastric adenocarcinoma?

- For patients with HER2 overexpression (defined as IHC 3+ or IHC 1+ and IHC 2+ confirmed by FISH positive), the addition of trastuzumab to the first-line chemotherapy regimen is recommended. EVIDENCE LEVEL I, RECOMMENDATION A.
- For treatment with trastuzumab, in addition to overexpression of HER2, the patient should have good *performance status* (ECOG 0–1), ejection fraction >50% verified on echocardiogram and absence of moderate or severe cardiovascular disease (such as recent infarction, unstable angina and congestive heart failure). LEVEL OF EVIDENCE I, RECOMMENDATION A.
- For the combination of trastuzumab with chemotherapy, capecitabine and cisplatin every 3 weeks are recommended. LEVEL OF EVIDENCE I, RECOMMENDATION A.
- Alternatively, the combination of trastuzumab with cisplatin and infusional 5-FU every 3 weeks is recommended. LEVEL OF EVIDENCE I, RECOMMENDATION A.
- Alternatively, the combination of trastuzumab with FOLFOX is recommended. EVIDENCE LEVEL III, RECOMMENDATION A.
- Regarding the combination of trastuzumab and FOLFOX and a dose of trastuzumab of 4 mg/kg every 14 days is recommended. EVIDENCE LEVEL IV, RECOMMENDATION B.
- Maintenance of trastuzumab after first-line progression is not recommended. EVIDENCE LEVEL V, RECOMMENDATION D.

Molecular targeted drugs were incorporated into the medical practice for the treatment of different types of tumours to improve the efficacy of cancer treatments, to reduce its toxicity and to individualise therapy. This irreversible trend is also present in the treatment of gastric adenocarcinoma.

The positive HER2 receptor has been reported in approximately 15%–20% of gastroesophageal cancers. Trastuzumab is a monoclonal antibody anti-HER2 receptor, with proven efficacy in breast cancer. A pivotal randomised phase III trial (ToGA) evaluated the role of trastuzumab in combination with cisplatin and fluoropyrimidine (infusional 5-FU or capecitabine) in patients with advanced gastric or GEJ tumours with HER2 overexpression [8]. In this trial, all patients provided samples for IHC and FISH tests to assess HER2 overexpression. It is important to note that the criteria for HER2-positive status in gastric adenocarcinoma differ from the criteria used for breast cancer [49]. A total of 594 patients were recruited. The final report showed a significant gain in OS for the group that received trastuzumab (13.9 months versus 11.1 months; HR = 0.74; 95% CI 0.6–0.91; *p* = 0.0046). In the subgroup with intense HER2 overexpression (IHC 3+ or IHC 2+ with positive FISH), OS gain was even higher, with a median of 16 months.

If the patient is a candidate to receive treatment with trastuzumab, the initial infusion should be 8 mg/kg in the induction phase every 3 weeks, followed by 6 mg/kg in the subsequent cycles for the maintenance phase.

So far, the only other anti-HER-2 therapy to prove efficacy in HER-2 positive metastatic gastric or EGJ adenocarcinoma was trastuzumab deruxtecan, an antibody-drug conjugate of trastuzumab, a cleavable tetrapeptide-based linker and a cytotoxic topoisomerase I inhibitor. A recently published phase II trial compared trastuzumab deruxtecan versus physician’s choice of chemotherapy for 187 patients who had progressed through at least two previous therapies, including trastuzumab. Both response rate (51% versus 14%, *p* < 0.001) and median overall survival (12.5 versus 8.4 months, *p* = 0.01) were significantly higher among patients receiving trastuzumab deruxtecan [79]. Nonetheless, this novel drug is yet not approved in Brazil.

#### When is ISH test necessary in patients with HER2-positive gastric cancer?

- The ISH test should be carried out only in patients with IHC 2+. LEVEL OF EVIDENCE I, RECOMMENDATION A.

#### What chemotherapy drugs are recommended for the second or more lines of chemotherapy in patients with advanced gastric cancer?

- Second-line chemotherapy should be recommended for patients with good *performance status* (ECOG 0–1). LEVEL OF EVIDENCE I, RECOMMENDATION A.
- Paclitaxel associated with ramucirumab, when available, is recommended for patients with ECOG 0–1. LEVEL OF EVIDENCE I, RECOMMENDATION A.
- Monotherapy with ramucirumab, when available, is recommended for patients with ECOG 0–1. LEVEL OF EVIDENCE I, RECOMMENDATION B.
- Monotherapy with irinotecan may be an alternative in the second or more lines of chemotherapy for advanced gastric cancer, depending on the regimen used in the first line. EVIDENCE LEVEL I, RECOMMENDATION B.
- Monotherapy with docetaxel or paclitaxel may be an alternative in the treatment of second and more lines of advanced gastric cancer, depending on the regimen used in the first line. EVIDENCE LEVEL II, RECOMMENDATION B.
- Third or more lines of chemotherapy may be used in patients with metastatic gastric cancer, depending on previous regimens and performance status. EVIDENCE LEVEL IV, RECOMMENDATION C.

In the past decade, phase III studies have defined the role of the second-line in metastatic gastric cancer. The German AIO study, which was closed prematurely due to low recruitment, was the first phase III trial to investigate second-line chemotherapy in advanced gastric cancer [115]. Patients with gastric or advanced GEJ adenocarcinoma previously treated with palliative chemotherapy were randomised to receive irinotecan or best supportive care. The dose of irinotecan was 250 mg/m² in the first cycle and, if well tolerated, was scaled to 350 mg/m² in subsequent cycles. The median OS was 4.0 months in the irinotecan group and 2.4 months in the placebo group (HR = 0.48, 95% CI 0.25–0.92; *p* = 0.012).

The second large phase III trial conducted in South Korea, which compared second-line chemotherapy with placebo in gastric cancer, was suspended in 2009 after the results of the AIO study, as discussed earlier. Palliative chemotherapy could be chosen at the discretion of the investigator (docetaxel 60 mg/m² every 21 days, or irinotecan 150 mg/m² every 2 weeks). The study also demonstrated a significant gain in median OS with chemotherapy (5.1 months versus 3.8 months; HR = 0.63 95% CI 0.47–0.86; *p* = 0.004) [92].

The Japanese phase III trial WJOG4007, presented at the ASCO 2012, compared irinotecan 150 mg/m² every 2 weeks versus paclitaxel 80 mg/m² weekly in patients refractory to fluoropyrimidine and platinum [116]. No difference was observed in OS, PFS and response rate between groups.

At the ASCO Gastrointestinal Cancer Symposium in 2013, two phase III trials showed positive results. The first trial (COUGAR-02) randomised patients with stomach, oesophageal or GEJ adenocarcinoma after progressing to fluoropyrimidine/platinum to receive docetaxel 75 mg/m² every 3 weeks or treatment for symptoms control [42]. The study demonstrated increased OS with the use of docetaxel (5.2 months versus 3.6 months; HR = 0.67; 95% CI 0.49–0.92; *p* = 0.01). The incidence of grade 4 toxicities with chemotherapy was 21%.

The second study (REGARD) evaluated the role of ramucirumab, a monoclonal antibody anti-VEGFR-2. Patients with progressing gastric or GEJ adenocarcinoma after first-line treatment with platinum or fluoropyrimidine or both received ramucirumab 8 mg/kg every 2 weeks or placebo [44]. The median OS was 5.2 months for the ramucirumab-treated group versus 3.8 months for the placebo group (HR = 0.776; 95% CI, 0.603–0.998; *p* = 0.0473). There was no impact on the response rate, and the toxicity profile was considered acceptable.

A meta-analysis of the three studies with irinotecan and docetaxel in second-line treatment was also published. A total of 410 patients were included, and the tested chemotherapy showed a significant reduction in the risk of death compared to clinical support (HR = 0.64; 95% CI 0.52–0.79; *p* < 0.00010) [58].

Patients with *NTRK* fusion-positive tumours of different primary sites had a response rate of 75% after treatment with larotrectinib. The progression-free survival rate at 1 year was 55%. Although exceptionally rare in oesophagogastric tumours, NTRK-fusion testing is recommended to identify eventual candidates to NTRK inhibitors [36]. Other NTRK inhibitors are not approved in Brazil.

Because of the possibility of effective second-line treatment, the course of treatment of metastatic gastric cancer has been changing, and an increasing number of patients are treated with sequential therapies. With this perspective, the personalisation of treatments is taking into account the predictive clinical factors that interfere with survival. Randomised clinical trials are essential to define the appropriate treatment sequence for metastatic gastric cancer and the role of biological agents. Other drugs with potential activity in later lines of therapy for metastatic gastric cancer, such as TAS-102 and regorafenib, are not approved in Brazil for this indication.

#### What is the role of immunotherapy in the treatment of advanced gastric cancer?

- Pembrolizumab may be used in the third-line or subsequent treatments of patients with ECOG 0 or 1 and PD-L1-positive gastric or EGJ adenocarcinoma. EVIDENCE LEVEL III, RECOMMENDATION A.
- Response to immunotherapy is lower in PD-L1-negative patients, but a small fraction of these patients benefit from the treatment. EVIDENCE LEVEL III, RECOMMENDATION C.
- Pembrolizumab is indicated for second-line or subsequent lines of treatment in patients with advanced gastric or EGJ adenocarcinoma and MSI, although this indication has not yet been approved in Brazil. EVIDENCE LEVEL III, RECOMMENDATION A.

Pembrolizumab is a selective and high-affinity monoclonal antibody anti-PD-1 and showed an objective response rate of 22% in a phase Ib clinical trial, KEYNOTE-012, that included 39 patients with advanced gastric or EGJ adenocarcinoma positive for PD-L1. Due to the promising results, the phase II KEYNOTE-059 trial investigated the safety and efficacy of pembrolizumab in 259 patients with metastatic gastric cancer who progressed after two or more lines of therapy. Patients received 200 mg of pembrolizumab every 3 weeks until disease progression or unacceptable toxicity. The objective response rate was 11.6% (95% CI: 8.0%–16.1%), and a complete response was observed in 2.3% of patients (95% CI: 0.9%–5.0%). Patients with positive and negative PD-L1 tumours showed objective response rates of 15.5% (95% CI: 10.1%–22.4%) and 6.4% (95% CI: 2.6–12.8%), respectively. One hundred and seventy-four patients (67.2%) were eligible for the evaluation of MSI, and seven participants were classified as MSI-high. Four participants with MSI-high tumours responded (response rate of 57.1%, 95% CI: 18.4%–90.1%), while the response rate among non-MSI-high patients was 9% (95% CI: 5.1%–14.4%). Results were consistent with other studies that observed an increased rate for PD-L1 amplification in tumours with MSI [9, 35]. Although representing a small portion of the participants (4%), a biomolecular profile requires further investigation. Regarding tolerability, 44 patients (17.8%) had at least one adverse event (Grade 3–5) relating to the treatment. Only two patients had their treatment discontinued due to adverse events and two treatment-related deaths were registered.

Le *et al*. [64] evaluated the activity of pembrolizumab in a cohort of 78 patients with advanced mismatch repair-deficient cancers across 12 different tumour types, from which five had gastroesophageal primaries. Objective responses were observed in 53% of patients. Responses were durable, with median progression-free survival and overall survival still not reached. The activity of pembrolizumab in MSI-H tumours gave the drug the first approval of a tissue-site agnostic agent by the FDA [64].

#### In which scenarios should curative-intent surgery be considered in the metastatic setting?

- The resection of liver metastasis may be considered in highly selected cases, such as single metastasis that can undergo R0 resection determined with high-quality image staging and absence of other metastasis, except regional lymph nodes, isolated retroperitoneal lymph nodes, good *performance status* (ECOG < 2), with a confirmed response to systemic treatment. These procedures should be conducted in experienced centers. Each case should be discussed in a multidisciplinary board. EVIDENCE LEVEL III, RECOMMENDATION C.
- In cases with peritoneal metastasis, no consensus was obtained from the board. Therefore, we suggest referring patients with a low PCI index (<9), who responded to systemic treatment, and with excellent *performance status* (ECOG 0-1), to reference centres (that manage >12 cases per year), with experience in this specific type of surgery to discuss its feasibility and indication.

#### In which scenarios should palliative surgery be considered?

- In patients with non-curable gastric cancer, chemotherapy is the standard treatment. EVIDENCE LEVEL I, RECOMMENDATION A.
- Limited gastric resections should be carried out only for palliative purposes of symptoms control. EVIDENCE LEVEL I, RECOMMENDATION B.
- Palliative (prophylactic) gastric resection has a limited role in increasing survival or in controlling symptoms, if compared to current chemotherapy. EVIDENCE LEVEL IV, RECOMMENDATION D.
- Surgery remains one of the most important alternatives to cases of obstruction, perforation or bleeding. However, its importance is reduced when effective systemic treatment, endoscopic interventions, interventional radiology and radiotherapy are available. In most cases, it is the last alternative of choice for the treatment of upper gastrointestinal bleeding related to the gastric tumour. EVIDENCE LEVEL IV, RECOMMENDATION B.

Gastric cancer is often diagnosed in an advanced palliative stage. At this point, although the patient is no longer a candidate for tumour resection with curative intent, palliative surgery is frequently considered. There are no randomised clinical trials assessing the benefits of palliative surgery in terms of OS. Several retrospective studies and case series have failed to demonstrate a survival benefit through palliative surgery, while others point to a potential advantage associated with surgery [23, 71, 112, 128]. All these studies, however, reported a high incidence of postoperative complications, with a potential impact on patients’ quality of life.

Before the surgical procedure, it is vital to identify patients who are at a higher nutritional risk. In a literature review conducted by Waitzberg *et al* [119], the nutritional support in reducing postoperative infection rates were analysed in 17 studies, with a total of 2,305 patients. It was observed that adequate nutritional support reduces the risk of postoperative infection from 61% to 39%. Also, the study observed a reduction in the hospital stay by two days, as well as a reduction in the occurrence of the anastomotic fistula by up to 46% [119].

The main symptoms requiring prioritisation in clinical management of patients with advanced gastric cancer are obstruction, bleeding, perforation and difficulties in nutritional support. Acute bleeding should be addressed by upper gastrointestinal endoscopy [53]. Surgery should be the last option in haemorrhagic conditions, even after haemostatic radiotherapy [60]. For the treatment of obstructive conditions, the endoscopic approach of balloon dilation with or without stent placement is preferred [57].

#### How should patients with gastric cancer be followed-up after treatment for curative purposes?

- In patients with non-invasive (*in situ*) carcinoma treated with endoscopic resection, UDE is recommended every 6 months for 1 year, and annually until the third year. CT of the chest and total abdomen can also be carried out, but only if clinically indicated. EVIDENCE LEVEL IV, RECOMMENDATION A.
- If clinically indicated, CT scans of the chest and total abdomen should be carried out, along with UDE in patients with non-invasive carcinoma treated with endoscopic resection. EVIDENCE LEVEL V, RECOMMENDATION A.
- For patients with T1 or T2 staging and both, N0, UDE and CT scans of the chest and total abdomen should only be carried out if clinically indicated. EVIDENCE LEVEL V, RECOMMENDATION A.
- For patients with T3 or T4 staging, whether N0 or N+, UDE should be carried out only if clinically indicated. EVIDENCE LEVEL V, RECOMMENDATION A.
- CT scans of the chest and total abdomen should be carried out every 6 months for the first 2 years, and annually until the fifth year in patients with T3 or T4 lesions, whether N0 or N+. EVIDENCE LEVEL V, RECOMMENDATION C.
- Additional tests, such as a complete blood count, iron determination and vitamin B12, may be carried out at the discretion of the physician to corroborate follow-up monitoring. EVIDENCE LEVEL V, RECOMMENDATION C.

## Conclusion

The scientific evidence must endorse decisions in daily medical practice. Cancer patients present complex requirements that are often difficult to be addressed by a single specialist. The GTG suggests a multidisciplinary approach and monitoring of patients with gastric cancer. In line with this, the GTG developed local specialist consensus guidelines on gastric cancer. Nonetheless, the therapeutic choice of the physician must always prevail, considering the individuality of the patient.

## Conflicts of interest

The authors declare that there is no conflict of interest.

## Funding source

There were no funding sources for this project.

## Figures and Tables

**Table 1.** Level of evidence (A) and strength of recommendation (B). CDC Classification System.

A) Level of evidence	
I	Evidence of at least one large randomised controlled trial (RCT) of good methodological quality (low potential for bias) or meta-analyses of well-conducted randomised clinical trials without heterogeneity
II	RCTs * small or large RCTs * with suspicion of bias (low methodological quality) or meta-analyses of these tests or tests with demonstrated heterogeneity
III	Prospective cohort studies
IV	Retrospective cohort studies or case-control studies
V	Studies without a control group, case reports, and expert opinions
* RCTs = Randomised clinical trials	
**B) Strength of recommendation**	
A	Solid evidence of efficacy with substantial clinical advantage—strongly recommended
B	Strong or moderate evidence, in terms of effectiveness, but with a limited clinical benefit—generally recommended
C	Insufficient evidence of effectiveness or benefit does not outweigh the risks/disadvantages (i.e., adverse events or costs)—optional
D	Moderate evidence against effectiveness or evidence indicating adverse outcomes—generally not recommended
E	Strong evidence against effectiveness or indicating adverse outcomes—not recommended

**Table 2.** Clinical syndromes and genes associated with hereditary gastric cancer.

Syndrome	Gene (s)	Clinical criteria suggesting this diagnosis	Gastric cancer risk	Risks for other cancers
Hereditary diffuse gastric cancer (HDGC)	CDH1	Diffuse gastric cancer (DGC) and an FH of one or more first- or second-degree relatives with GC; or personal and/or FH of DGC diagnosed before the age of 40; or personal and/or FH of DGC and LBC, one diagnosed before the age of 50. Obs.: the majority of gastric cancers are of the diffuse type.	70%–80%	Breast (lobular)
Juvenile polyposis (JPS)	*SMAD4* *BMPR1A*	Presence of five or more juvenile polyps in the colorectum or any juvenile polyps in other parts of the GI tract.	20%–30%	Duodenal, colorectal
Peutz-Jeghers (PJS)	*STK11*	Presence of perioral or buccal hyperpigmented spots and/or two or more histologically characteristic GI hamartomatous polyp(s) or a solid tumour typical of PJS or a family history of the syndrome.	29%	Breast (any), ovary, colorectal, pancreatic.
Lynch (LS)	*MLH1 MSH2 MSH6 PMS2* *EPCAM*	GC with personal or FH consistent with LS (Amsterdam or Bethesda criteria [20]; Tumour with mismatch repair deficiency detected by IHC or MSI testing; Known family pathogenic variant in a LS gene;Risk of ≥5% chance of LS based on risk prediction models; Obs.: the majority of GC in LS are of the intestinal type.	5%–13%	Duodenal, colorectal, ovarian, endometrial, urinary tract, pancreatic
Li-Fraumeni (LFS)	*TP53*	Personal or FH consistent with the Chompret criteria for LFS [104].	1%–4%	Breast (any), CNS, sarcomas, lung, adrenal cortical and other.
Familial adenomatous polyposis (FAP)	*APC*	Presence of ≥100 synchronous colorectal adenomas (“classic” FAP) or 10–99 synchronous adenomas (attenuated FAP).	0.6%	Duodenal, colorectal, thyroid
Cowden (CS)	*PTEN*	Presence of multiple GI hamartomas or ganglioneuromas and a group of major and minor criteria including other solid tumours and non-tumoural findings [39]	Increased, but exact % not determined	Breast (any), endometrial, thyroid.

**Table 3.** Ryan’s modified system for therapeutic response.

Description	Score
No viable cancer cells (complete response)	0
Isolated cells or rare small groups of cancer cells (almost complete response)	1
Residual cancer with evident tumour regression, however, with more than a single cell or rare small groups of cancer cells (partial response)	2
Extensive residual cancer without evident tumour regression (low or no response)	3
